# Effect of a Single Simulated 500 m Saturation Dive on Lung Function

**DOI:** 10.3389/fphys.2022.911167

**Published:** 2022-06-02

**Authors:** Ningfang Lian, Sijiao Wang, Lijuan Hu, Liping Xue, Ying Gong, Li Li, Yanjie Yang, Lei Zhu

**Affiliations:** ^1^ Department of Pulmonary and Critical Care Medicine, The First Affiliated Hospital of Fujian Medical University, Institute of Respiratory Disease, Fujian Medical University, Fujian, China; ^2^ Department of Pulmonary and Critical Care Medicine, Zhongshan Hospital, Fudan University, Shanghai, China; ^3^ Critical Care and Respiratory Care Department, Beijing Tsinghua Changgung Hospital Affiliated to Tsinghua University, Beijing, China

**Keywords:** saturation diving, lung function, pulmonary diffusion function, forced vital capacity, forced expiratory volume in 1 s

## Abstract

**Background:** Whether deep saturation diving causes injury to lung function remains controversial and the mechanism is unclear. The present study aimed to evaluate the effects of a 500 m simulated single saturation dive on lung function.

**Methods:** A retrospective study was performed in nine professional divers who spent 176 h in a high-pressure environment simulating a depth of 500-m saturation dive (51 atm, 5.02 Mpa). Pulmonary function parameters were investigated and compared before and on 3 days after the dive.

**Results:** Nine professional divers aged (36 ± 7) years were enrolled. Three days after the dive, the parameters related to expiratory flow (forced expiratory volume in 1 s (FEV_1_)/forced vital capacity (FVC)) were decreased; the parameters related to small airway function (forced expiratory flow at 50%, 75% of FVC exhaled and forced mid-expiratory flow) were decreased compared with those before the dive (both *p* < 0.05). Additionally, after the dive, the parameters related to pulmonary diffusion function were decreased compared with those before the dive (both *p* < 0.05). The parameters related to lung volume (residual volume, vital capacity and total lung volume) and those related to respiratory exertion (peak expiratory flow and forced expiratory flow at 75% of FVC exhaled) were not significantly different between after and before the dive. Two divers with small airway dysfunction before the dive had obstructive ventilatory dysfunction after the dive. Additionally, mild obstructive ventilatory dysfunction in three divers before the dive became severe after the dive. After a bronchial dilation test, five divers showed improvement of FEV_1_, which ranged from 0.10 to 0.55 L. Chest radiographs and echocardiography of all divers were normal after diving.

**Conclusion:** 500 m simulated saturation diving induces a decrease in small airway function and diffusion function. This injury may be associated with small airway and diffusion membrane lesions.

## Introduction

Saturation diving is a diving method that allows people to be directly exposed to high-pressure environments to achieve long-term and large-depth work. It is also a cutting-edge technology for human beings to challenge ocean space and physiological limits. The characteristic of saturation dives are high density of the breathing gas and high ambient pressure. In the traditional view, a high density of the breathing gas and a high ambient pressure are the leading cause of lung function defects in divers (Skogstad et al., 1996; Tetzlaff and Thomas, 2017). Lung function defects recover soon after the end of the diving. A previous study showed no significant changes in lung function after 1–2 days and 6–8 weeks after a helium–oxygen mixed gas saturation dive to 2.5 MPa (Thorsen et al., 2006). However, in other studies, the divers still had lung function defects a long time after they finished diving. Lehnigk et al. found that a single saturation dive of 360 or 450 m impaired gas exchange at 0, 24, and 48 h after the dive (Lehnigk et al., 1997). Thorsen et al. showed that 5–450 m of sea water saturation diving decreased mid-expiratory flow and pulmonary diffusion function (Thorsen et al., 1994). However, the sample size of these studies was limited. Previous reported saturation diving depths of 5–450 m did not reflect the effects of deeper saturation diving on lung function. Some data were also not systematic and complete, which affected the research conclusions. Therefore, whether deep saturation diving causes injury to lung function remains controversial and the mechanism is unclear.

The 500 m saturation diving land-based manned experiment is the deepest simulated saturated diving test ever reported in China. During the experiment, the divers entered a pressurized chamber on land to simulate the deep ocean. The ambient pressure at depths of 500 m of sea water, corresponding to absolute pressures of 5.02 MPa. The present study aimed to evaluate the effects of a 500 m simulated single saturation dive on lung function.

## Methods

This retrospective study was conducted in the Pulmonary Function Room of Zhongshan Hospital, Fudan University. Nine divers who completed a 500 m simulated saturation dive in June 2021 were enrolled in the study.

The divers began to exercise 2 months before the diving test, and the amount of exercise was set according to the tolerance required in the diving process. The training predive included: 1) Physical training:jogging and other morning exercises, swimming pool training in the afternoon and strength training in the gym in the evening. 2) Specific training: Each diver underwent 20 times progressive compression and decompression exercises. After that, the diver completed a fitness test in a high-pressure environment. The schedule of diving included compression time 175 h, bottom time 176 h and decompression time 21 days. The pressurization rate slowed down as the simulated depth of the chamber increased. Each of the nine divers spent 176 h in a high-pressure environment simulating a depth of 500 m (51 atm, 5.02 Mpa) to complete various experiments. The breathing atmosphere was a mixture of oxygen and helium with the PO_2_ at 0.4 bar.

Lung function tests were performed 1 day before diving and 3 days after the dive. Chest radiographs and Doppler echocardiography were performed 3 days after dive. The local ethics committee approved the study protocol.

The weight and height of the divers were measured while wearing indoor clothing. Lung function were investigated with an MS-PFT Spirometer (Jaeger, Germany). A trained operator demonstrated the required maneuvers to the divers before the tests. We recorded and analyzed the following pulmonary function parameters: forced vital capacity (FVC), forced expiratory volume in 1 s (FEV_1_), FEV_1_/FVC, peak expiratory flow (PEF), forced expiratory flow at 25, 50, and 75% of exhaled FVC (FEF_25_, FEF_50_, and FEF_75_), mid-expiratory flow (FEF25-75%), diffusion capacity of the lungs for carbon monoxide (DLCO), DLCO/unit of alveolar volume (DLCO/VA), residual volume (RV), vital capacity (VC), and total lung capacity (TLC). DLCO and TLC were determined by one breath dispersion method.

The detection of pulmonary function parameters and interpretation were followed in accordance with American Thoracic Society/European Respiratory Society (ATS/ERS) standardization (Culver et al., 2017; Graham et al., 2019). Obstructive ventilatory defect was defined as reduced FEV_1_/FVC lower than lower limit of normal. The severity were defined by FEV_1_%pred (FEV_1_ measured value/FEV_1_ predicted value); mild (FEV_1_%pred > 70%), moderate (60% ≤ FEV_1_%pred < 69%), moderately severe (50% ≤ FEV_1_%pred < 59%), severe (35% ≤ FEV_1_%pred < 49%), and very severe (FEV_1_% < 35%pred) (Pellegrino et al., 2005). If the subject did not meet the criteria of obstructive ventilatory defect, FEF25-75%, FEF 50% and FEF75% were used to evaluate the small airways function. when two of the above three indicators are below 65% prediction values, small airway dysfunction was defined (Graham et al., 2019).

Bronchodilation tests were performed on divers with obstructive airway dysfunction. At 15–20 min after inhaling salbutamol, a percentage change ≥12% and an absolute change ≥200 ml in FEV_1_ compared with pre-bronchodilator values were considered as a positive bronchodilation test.

Statistical analysis and graphing were performed using GraphPad Prism software, version 7.0 (GraphPad Software, United States). The Kolmogorov–Smirnov test was used to analyze the normality of data. The paired *t*-test was used to compare the parameters. *p* < 0.05 was considered statistically significant.

## Results

Nine experienced saturation divers were included in this study. The mean age of the divers was 36 ± 7 years (range: 31–5 years), and all the divers were men. The mean height was 176 ± 7 cm and the mean weight was 80 ± 11 kg.

### Pre-Dive Lung Function

The ventilatory function of all divers was appropriate before the dive. The mean values of FVC and FEV_1_ were (5.33 ± 0.61) L and (4.16 ± 0.51) L, respectively. The mean percentages of the predicted values of FVC and FEV_1_ were 113 ± 10.58% and 106 ± 11.03%, respectively. Among the divers, three (33.3%) had mild obstructive ventilatory function defect and two (22.2%) had small airway dysfunction. These three divers with mild obstructive ventilatory function defect had bronchodilation test. Absolute change in FEV_1_ ranged from 0.15 to 0.33 L, and percentage change in FEV_1_ varied from 3.54 to 9.30%. All had a negative bronchial dilation test.

All (100.0%) divers had a normal DLCO and DLCO/unit of alveolar volume before the dive (Table 1).

**TABLE 1:** Lung function test results from nine divers before dive.

Divers	Ages (ys)		Height (cm)	Weight (kg	FEV_1_	FVC	FEV_1_/FVC	PEF	FEF_25_	FEF_50_	FEF_75_	RV	TLC	RV/TLC	DLCO	DLCO/VA
1	41	171		80	98.4	116.8	69.28	93.00	65.40	58.50	52.60	133.53	113.70	133.53	132.53	106.35
2	34	177		91	103.7	122.8	69.97	97.50	73.10	64.50	56.40	152.49	123.80	152.49	118.59	84.42
3	33	174		74	103.4	117.8	74.03	103.10	96.30	63.50	49.70	118.81	112.14	118.81	106.06	86.63
4	28	182		95	85.0	91.0	77.44	89.01	78.89	63.53	56.89	102.46	89.02	102.46	107.64	105.68
5	53	189		90	111.1	122.3	74.52	125.36	113.68	85.05	53.57	131.38	119.91	131.38	129.12	93.26
6	31	179		67	124.1	119.8	89.18	126.43	124.69	140.84	109.18	108.53	111.53	108.53	138.82	114.38
7	34	175		83	115.8	116.2	83.35	122.25	135.83	111.04	89.05	120.33	113.65	120.33	107.85	84.97
8	34	170		63	108.6	112.4	82.48	130.14	107.78	108.54	77.04	101.72	103.88	101.72	98.27	90.35
9	35	167		78	103.9	101.6	85.06	113.86	135.70	134.88	83.29	83.46	93.91	83.46	94.48	94.51

a Lung function test parameters are presented as percentage of measured/predicted values, except for FEV1/FVC; Abbreviations: FVC, forced vital capacity; FEV_1_, forced expiratory volume in 1 s; PEF, peak expiratory flow; FEF_25_, EF_50_, and FEF_75_, forced expiratory flow at 25, 50, and 75% of exhaled FVC; DLCO, diffusion capacity of the lungs for carbon monoxide; DLCO/VA, DLCO/unit of alveolar volume; RV, residual volume; VC, vital capacity; TLC, total lung volume.

### Lung function Before and After the Dive

**FIGURE 1:**
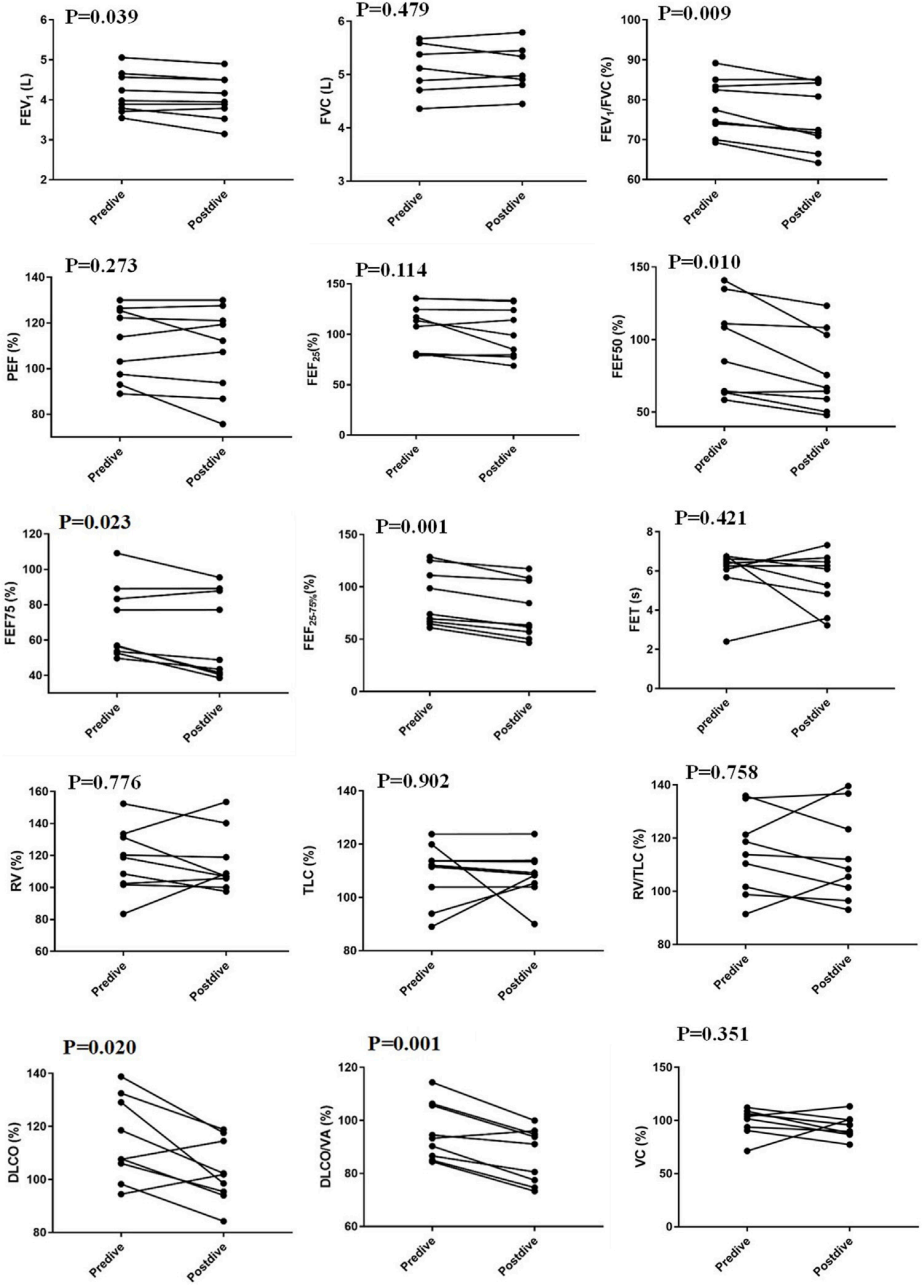
Changes in pulmonary function parameters between 3 days after a simulated dive and before the dive. FVC, forced vital capacity; FEV1, forced expiratory volume in 1 second; PEF, peak expiratory flow; FEF25, FEF50, and FEF75, forced expiratory flow at 25%, 50%, and 75% of exhaled FVC; DLCO, diffusion capacity of the lungs for carbon monoxide; DLCO/VA, DLCO/unit of alveolar volume; RV, residual volume; VC vital capacity; TLC, total lung capacity; FET, forced expiratory time.

Three days after the simulated 500 m saturation dive, the nine divers performed a lung function test again. FEF_50_, FEF_75_, FEF_25–75%_, FEV_1_/FVC, FEV_1_, DLCO, and DLCO/VA were significantly decreased 3 days after the dive compared with those before the dive (all *p* < 0.05, Table 2). There were no significant differences in volume parameters (VC, RV, TLC, and RV/TLC) between after and before dive (Figure 1). FVC, PEF, and FEF_25_ were also not different between after and before the dive. A diver showed a large decrease in TLC (from 120 to 90% of the predicted value), while two divers showed a rather large increase (from 89 to 108%, from 94 to 105% of the predicted value) (Figure 1). Chest radiographs and Doppler echocardiography of all divers were normal after diving.

**TABLE 2:** Changes in pulmonary function parameters between 3 days after the simulated dive and before the dive.

	Predive	3 days after Dive	*p* Value
FEV1 (L)	4.16 ± 0.51	4.04 ± 0.54	0.039
FVC (L)	5.33 ± 0.61	5.34 ± 0.65	0.479
FEV1/FVC	78.4 ± 7.0	75.6 ± 8.2	0.009
PEF (%)	111 ± 16	108 ± 19	0.273
FEF_25_ (%)	103 ± 27	95 ± 32	0.114
FEF_50_ (%)	92 ± 32	77 ± 27	0.010
FEF_75_ (%)	70 ± 21	63 ± 24	0.023
FEF_25-75_ (%)	89 ± 27	77 ± 27	0.001
FET(s)	5.9 ± 1.4	5.5 ± 1.4	0.421
RV (%)	117 ± 21	115 ± 19	0.776
TLC (%)	109 ± 11	108 ± 9	0.902
RV/TLC (%)	114 ± 15	112 ± 17	0.758
DLCO(%)	115 ± 16	103 ± 12	0.020
DLCO/VA (%)	96 ± 11	87 ± 10	0.001
VC(%)	113 ± 11	114 ± 10	0.351

a Lung function test parameters are presented as percentage of measured/predicted values, except for FEV_1_/FVC; Abbreviations: FVC, forced vital capacity; FEV_1_, forced expiratory volume in 1 s; PEF, peak expiratory flow; FEF_25_, FEF_50_, and FEF_75_, forced expiratory flow at 25, 50, and 75% of exhaled FVC; DLCO, diffusion capacity of the lungs for carbon monoxide; DLCO/VA, DLCO/unit of alveolar volume; RV, residual volume; VC, vital capacity; TLC, total lung capacity.

Three days after the dive, two divers with small airway dysfunction before the dive developed into mild obstructive ventilation defect; three divers with mild obstructive lung function defect before the dive still had mild obstructive lung function defect, the FEV_1_%pred average of three divers decreased from (86.7 ± 1.9)% to (82.1 ± 3.7)%. After a bronchial dilation test, divers with obstructive ventilatory dysfunction showed an improvement in FEV_1_. Absolute change in FEV_1_ ranged from 0.10 to 0.55 L, and percentage change in FEV_1_ varied from 2.53 to 17.46%. Two (40%) divers had a positive bronchial dilation test. After bronchodilation test, 3 (60%)divers returned to pre-dive FEV_1_ levels.

## Discussion

We report the effect of simulating a 500 m saturation dive on lung function. We found that the divers showed decreased small airway function and decreased diffusion function.

In this study, the divers still had impaired lung function 3 days after diving, which is in contrast to previous studies (Thorsen et al., 2006). This finding suggests that, even though the divers had a normal density of the breathing gas and a normal ambient pressure, the injury to lung function was not fully recovered by 3 days after the dive. Therefore, the injury cannot be entirely attributed to a high density of the breathing gas and high ambient pressure.

revious studies have shown decreased FEV_1_/FVC in divers who work at depths up to 340 m (Crosbie et al., 1979). In our study, a 500 m simulated saturation dive also resulted in a decrease in FEV_1_/FVC. One explanation for the decreased FEV_1_/FVC was associated with the increase in FVC. Several studies have shown that diving training contributes to a large VC (Crosbie et al., 1979; Thorsen et al., 1993; Thorsen et al., 1994). VC and FVC did not significantly change between before and after diving in our study, which indicated that the divers in our study had fully simulated pre-dive training, and that pre-dive breathing work had been increased to the same level as that during the simulated dive. Therefore, the decrease in the FEV_1_/FVC ratio in this study was not related to the changes in FVC. Additionally, in our study, force-dependent parameters (PEF and FEF_25_) did not significantly change before and after diving, which reflect that the lung function parameters were comparable before and after diving. Previous study found the decrease of FVC after deep sea dives using a closed-circuit Rebreather (CCR) (Dugrenot et al., 2021), which was different from our result. There were important differences between these two study. The diving depths of the two studies varied widely, at 500 m and around 100 m respectively. The 100-m dive is relatively easy to complete; while 500 m saturated diving is very difficult because of the huge variation in pressure of alveolar gas and tissue dissolved gas. The decompression was slower and performed in a dry chamber in our study, while CCR dives decompression was in water and relatively rapid. The partial pressure of oxygen was 0.4 bar (40 kpa) during saturation dives in our study, against 120–160 kPa in CCR dive. Different partial pressure of oxygen produced different concentrations of reactive oxygen species, which may be the reason for the increase of extravascular lung water during CCR diving. Therefore, hyperbaric oxygen during CCR diving may induce a decrease in FVC, but have little effect on saturated diving.

In this study, the pulmonary function parameters reflecting small airway function (FEF_50_, FEF_75_, and forced mid-expiratory flow) were decreased 3 days after the dive compared with those before the dive. The simultaneous reduction in FEV_1_/FVC and end-expiratory flow were an indication of obstructive of changes in the airway. In our study, the divers with a small airway dysfunction before the dive developed obstructive ventilatory dysfunction after the dive. Additionally, the divers with mild obstructive ventilatory dysfunction before the dive showed decreased of FEV1%pred after the dive, which support the obstructive airway changes either. Excluding the effect of a change in lung volume and perceived exertion, we speculate that the small airway dysfunction may have been related to small airway lesions during the deep dive.

In previous study, saturation diving reduced lung diffusion capacity. Lehnigk found normal DLCO, elevated VA, and decreased DLCO/VA after a single saturation dive of 360 m or 450 m^4^. The reduction in DLCO/VA after the dive may have been due to an elevation in VA (Sames et al., 2018). In our study, the 500 m simulated saturation dive decreased both DLCO and DLCO/VA. The divers in this study had normal lung function or only a mild obstructive lung function defect before the dive. None of the divers had any significant differences in lung volume before and after diving. Additionally, no pulmonary vascular diseases were found in any of the divers by echocardiography. The decrease in diffusion function could have been due to a lesion of diffusion membranes.

Usually, the small airway lesions and the diffusion membranes lesion was synchronous with lung parenchyma injury. In this study, the pressure of oxygen in breathing atmosphere was dynamically set at about 0.4 bar during the diving. In this situation, the incidence of oxygen poisoning was rare. At high ambient pressure, the amount of oxygen free radicals increased. It was not clear whether the increase of oxygen free radicals induced lung parenchymal injury, which can be clarified in future studies. Pulmonary oxygen toxicity (POT) index in our study we calculated using Equation (t^2^ × PO_2_^4.57^, where t is the time in h, and PO_2_ is the partial pressure of oxygen in bar) was 470 (Arieli, 2019). The levels of POT was associated with the duration of lung function impairment after saturated diving. The higher level of POT may be the reason for the small airway lesions and the diffusion membranes lesion 3 days after diving in our study.

A bronchial dilation test in five divers with obstructive ventilator dysfunction 3 days after the simulated dive showed varying degrees of improvement in FEV_1_. This finding suggested that peripheral airway lesions may include spasm of airway smooth muscle, but this is not completely reversible. Bronchodilators may be an effective method of ameliorating lung function defects induced by deep saturation diving.

TLC was indirectly detected by one breath dispersion method, which was influenced by many factors. In subjects with no or mild obstructive lung function defect, the trend of FVC, VC and TLC was consistent. VC and FVC were determined directly with good reliability. So, the reliability of research conclusions is not affected.

There are some limitations to this study. First, because only nine divers participated in the 500 m saturation dive, the limited sample size may have affected the reliability of the conclusions. Second, because of the limitations of research ethics and research conditions, a pulmonary function test was not performed underwater, which would directly reflect the effect of saturation diving on lung function. Finally, we hypothesized that deep saturation diving induced small airway and diffusion membrane lesions, which is just based on lung function tests. However, because of ethical limitations, no pathological examination was conducted.

In conclusion, deep saturation diving induced a decrease in small airway function and diffusion function. The injury maybe associated with small airway and diffusion membrane organic lesion.

## Data Availability

The raw data supporting the conclusion of this article will be made available by the authors, without undue reservation.
